# Impact of Dental Anxiety on Dental Care Routine and Oral-Health-Related Quality of Life in a German Adult Population—A Cross-Sectional Study

**DOI:** 10.3390/jcm12165291

**Published:** 2023-08-14

**Authors:** Christian H. Winkler, Monika Bjelopavlovic, Karl M. Lehmann, Katja Petrowski, Lisa Irmscher, Hendrik Berth

**Affiliations:** 1 Department of Prosthetic Dentistry, University Medical Center of the Johannes Gutenberg-University Mainz, Augustusplatz 2, 55131 Mainz, Germany; christian.winkler@unimedizin-mainz.de (C.H.W.); karl.lehmann@unimedizin-mainz.de (K.M.L.); 2 Department of Medical Psychology and Medical Sociology, University Medical Center of the Johannes Gutenberg-University Mainz, Duesbergweg 6, 55131 Mainz, Germany; kpetrows@uni-mainz.de; 3 Division of Psychological and Social Medicine and Developmental Neurosciences, Research Group Medical Psychology and Medical Sociology, Medical Faculty Carl Gustav Carus, Technische Universität Dresden, Fetscherstr. 74, 01307 Dresden, Germanyh.berth@ukdd.de (H.B.)

**Keywords:** dental anxiety, dental care, dental anxiety score, oral health impact profile, oral health

## Abstract

The interaction between dental anxiety and the establishment of a consistent dental care routine has been an ongoing challenge. Unfortunately, there is limited available data concerning the detailed dental care practices of individuals with dental anxiety. Therefore, this study aims to explore how dental anxiety influences dental care habits and oral-health-related quality of life within an adult population. By utilizing the Dental Anxiety Scale (DAS) and the German Oral Health Impact Profile (OHIP-G5), we assessed their extent. To evaluate the differences, we performed analyses of variance (Anova), an independent *t*-test and rank correlation. The findings of this study unveil a significant correlation between elevated DAS scores and reduced frequency of tooth brushing; calculus removal and appointments for professional teeth cleaning. Interestingly; the use of dental floss and mouthwash solution as well as toothbrush hardness appeared to be not significantly affected by dental fear. Moreover, individuals with dental anxiety demonstrated a preference for manual toothbrushes over electric ones. In addition, higher DAS scores were found to be strongly associated with greater OHIP-G5 scores, thus leading to a substantial decline in overall oral health-related quality of life.

## 1. Introduction

Dental anxiety or fear is a widespread psychological phenomenon that affects a substantial portion of the population worldwide, often leading to avoidance or hesitation in seeking dental care [1,2,3,4]. It is characterized by feelings of apprehension, unease or fear specifically related to dental procedures and clinics [5]. This state of anxiety can vary in intensity, ranging from mild uneasiness to severe phobia, and it significantly affects an individual’s oral health and overall well-being. Dental care routine plays a vital role in maintaining optimal oral health, preventing dental diseases and ensuring an overall better quality of life as well as social acceptance. However, individuals with dental anxiety may experience considerable challenges in adhering to their daily dental care practices. The anxiety surrounding dental procedures and settings can give rise to avoidance behaviors, resulting in poor oral hygiene, delayed dental treatment, and compromised oral health outcomes. This can perpetuate a vicious cycle of shame, deteriorating oral health, and further avoidance of necessary treatments [6,7]. Dental anxiety and avoidance behavior can manifest early in children, significantly influenced by external factors, particularly within the family environment [8].

“Dental anxiety, a prevalent concern, is rooted in a complex interplay of cognitive and non-cognitive factors, which have been extensively investigated in the literature [9,10,11]. Cognitive factors encompass an individual’s thoughts, beliefs, and perceptions linked to dental encounters. Negative beliefs pertaining to pain, perceived loss of control, or prior traumatic experiences have been identified as contributors to the origination and endurance of dental anxiety. Consequently, the adoption of strategies such as the tell-show-do technique is recommended during patient treatment, fostering a sense of control [12]. Non-cognitive factors involve emotional and physiological dimensions. These encompass aversion to needles, apprehension of the clinical environment, and anticipation of discomfort. A study by Gasparro et al. delved into cognitive vulnerability patterns, investigating their variance based on factors such as gender and age, among others. This research highlighted the significance of an individual’s cognitions and perceptions in the context of dental treatment and, furthermore, could observe that a higher level of dental anxiety can be found in females [11]. This sociodemographic finding is supported in the literature [13,14].

Over the past few decades, oral health has made remarkable advancements, thanks to improvements in dental care, oral hygiene practices, and preventive measures, as evidenced by nationwide surveys [15]. These positive developments have led to improved oral health outcomes for the general population. However, recent studies have indicated that especially people with dental anxiety are barely benefiting from this positive development [2,7,16]. Even today, approximately 80% of adults in developed countries experience unease before dental treatment, around 20% feel scared of it, and roughly 5% completely avoid it [4]. Dental anxiety not only causes distress for patients but also presents challenges for dentists, leading to reduced cooperation, extended treatment durations, and the potential for misdiagnosis and mistreatment, such as inaccurate tooth vitality analysis [17,18]. Gaining a comprehensive understanding of how dental fear influences individuals’ dental care routines holds significant importance. By examining the factors contributing to dental fear and exploring its consequences on oral health behaviors, oral health professionals can implement customized approaches to mitigate the negative effects and enhance oral health outcomes.

Previous studies examining dental care within the context of dental anxiety have primarily focused on individual factors such as knowledge of oral hygiene, frequency of toothbrushing, and avoidance of dental visits. The majority of international studies consistently report a correlation between higher levels of dental anxiety and poorer oral hygiene practices [19,20,21,22]. Similar results were found in studies conducted in Germany, which observed corresponding outcomes for toothbrushing, dental flossing, and avoidance behavior [23,24]. In a descriptive study from India involving 100 college students, many dental care practices were examined individually. They observed that, as the frequency of brushing and mouthwash usage increased, the levels of dental anxiety among the participants decreased [25]. In contrast, a recent study in Israel found a negative association between dental anxiety and dental neglect [26]. However, there are also studies from Norway that could not demonstrate any significant differences in the frequencies of brushing and dental flossing [27]. To date, there is a lack of large-scale studies in the German population specifically investigating the impact of dental anxiety on common dental hygiene practices. The objective of this cross-sectional study is to explore the dental care habits among adults in Germany who experience dental anxiety as well as the impact of dental anxiety on oral-health-related quality of life. Based on prevailing results that people with dental anxiety are more likely to neglect dental care, we hypothesize that lower frequencies of brushing, flossing, mouthwash usage, as well as calculus removal and professional teeth cleaning will be associated with higher mean Dental Anxiety Scale (DAS) scores. We also anticipate that higher anxiety will be correlated with the choice of a soft toothbrush and the avoidance of an electric toothbrush. Our final assumption is that, with an increasing DAS, OHIP-G5 scores also increase, indicating a worse oral-health-related quality of life.

## 2. Materials and Methods

The objectives of this cross-sectional study were achieved through the collection of data from sixteen questionnaire surveys conducted by the Medical Faculty for Medical Psychology and Medical Sociology at the Technical University of Dresden between 2013 and 2019. These surveys were conducted either directly at the university or within its local vicinity. A total of N = 2666 questionnaires were subjected to statistical analysis. To be eligible for participation, individuals needed to be of legal age, have a sufficient understanding of the German language, and have the physical and mental capacity to complete the questionnaires. All participants included in the study provided written informed consent. In addition to sociodemographic data, the main aspects of this study were self-assessment of dental anxiety, oral-health-related quality of life, daily dental routine, and routine of dental prophylaxis appointments. Questions about dental routine included the frequency of daily brushing (never, once, twice, three times, or more), frequency of flossing and mouthwash (never, once a week, once a day, twice, or more a day), toothbrush hardness (soft, medium, hard) and the utilization of electric toothbrushes (yes, no). Furthermore, participants were asked about the annual frequency of calculus removal (never, once, twice, three times, or more) and if they attend professional teeth cleaning appointments (yes, no). For the assessment of dental anxiety and oral-health-related quality of life, the Dental Anxiety Scale (DAS) and the Oral Health Impact Profile (OHIP) were employed. The average age of participants was determined to be M = 45.5, with a standard deviation of SD = 18.2. In terms of gender distribution, 58.6% were female and 41.4% were male. Regarding marital status, 53.5% were single, while 46.5% indicated being in a partnership.

### 2.1. Instruments

#### 2.1.1. Dental Anxiety Scale

The Dental Anxiety Scale (DAS) is a questionnaire for self-assessment of dental treatment anxiety. It was the first scientifically validated measuring instrument for dental treatment anxiety and continues to be regularly used in studies [28,29]. The DAS consists of four questions, each representing a situation during a dental visit. Individuals are asked to prospectively imagine themselves in each described situation and indicate their level of anxiety on a five-point scale. The scale ranges from 1 = “relaxed” to 5 = “so anxious that it feels sick.” By evaluating all four responses, total scores ranging from 4 to 20 are obtained.

A score of up to 10 points indicates little to no anxiety. Scores between 11 and 15 suggest an anxious patient, while scores of 16 or higher are categorized as dental treatment phobia according to Corah and should be approached with caution by the dentist [29]. Due to its simplicity and brevity for patient completion, the Dental Anxiety Scale is a useful tool for initial screening in dental practices. To keep the questionnaire as short and simple as possible the shorter original form of Corah’s Dental Anxiety Scale was chosen over the modified five question Version (mDAS).

#### 2.1.2. Oral Health Impact Profile

The Oral Health Impact Profile (OHIP) is recognized as one of the most widely used and internationally comparable instruments for assessing oral-health-related quality of life. The OHIP aims to capture various aspects of oral complaints and the frequency of experiencing these aspects over the past month. When completing the OHIP questionnaire, respondents are presented with five response options for each item, which represent the frequency of occurrence: 0 = “never”, 1 = “hardly ever”, 2 = “occasionally”, 3 = “often”, and 4 = “very often”. By selecting the most appropriate response for each item, individuals provide insights into the impact of oral health on their overall quality of life. The original version of the OHIP developed in Australia consisted of 49 items, grouped into seven subscales. However, in this study, a shortened form known as OHIP-G5, developed in Germany, was utilized [30]. Despite the reduction in the number of items, the OHIP-G5 still captures 90% of the information that can be obtained from the original OHIP questionnaire [31]. The OHIP-G5 includes only five questions, allowing for a more efficient assessment of oral health-related quality of life. The scores obtained from the OHIP-G5 range from 0 to 20, where higher scores indicate a greater impact of oral health on one’s quality of life, and lower scores suggest fewer restrictions or limitations.

### 2.2. Statistical Analyses

The statistical software IBM SPSS Statistics V27 was utilized for analyzing the data obtained in this study. A significant level of *p* ≤ 0.05 was established to determine statistical significance. Prior to conducting the analysis, the homogeneity of variance was assessed using the Levene test. If the resulting *p*-value was greater than 0.05, it indicated homogeneity of variance. Conversely, if the *p*-value was less than 0.05, the assumption of variance equality was rejected. Based on these findings, we selected an appropriate test. When comparing the means of exactly two groups and the homogeneity of variance was confirmed, an independent samples *t*-test was employed. If homogeneity of variance could not be assumed, Welch’s *t*-test was used instead. To evaluate the strength of association, Cohen’s d was calculated, with values ranging from 0.2 to 0.5 indicating a small effect, 0.5 to 0.8 indicating a medium effect, and over 0.8 indicating a strong effect [32]. To examine the central tendencies of means between more than two groups, assuming homogeneity of variance, a one-way analysis of variance (ANOVA) was conducted. Post-hoc analysis was performed using the Tukey method. In cases where the Levene test indicated non-homogeneity of variance, Welch’s ANOVA was applied. The Games-Howell method was used for the post-hoc analysis. To assess the effect size, eta squared was calculated. Values greater than 0.01 indicated a weak effect, greater than 0.06 indicated a medium effect, and greater than 0.14 indicated a strong effect [32,33]. To analyze the impact of dental anxiety levels on oral-related quality of life, a rank correlation using Spearman’s correlation coefficient was employed to determine significance and effect size. Mean OHIP-G5 scores were calculated for each level of DAS score ranging from 4 to 20. We used Spearman’s Rho to assess the strength of the relationship, ranging from −1 to +1, with 0 indicating no connection between the two variables.

## 3. Results

### 3.1. Impact of Dental Anxiety on Daily Dental Hygiene

Table 1 displays mean dental anxiety levels linked to different tooth brushing frequencies. Brushing twice a day was overwhelmingly preferred (N = 1325). No brushing is associated with significantly higher anxiety. As brushing frequency rises, mean DAS scores generally decrease, but without statistical significance in the post-hoc Tukey analysis (eta^2^ = 0.08, medium effect size). Table 2 shows a notable divergence in dental treatment anxiety between electric toothbrush users and non-users. Higher DAS scores correspond to less electric toothbrush use (Cohen’s d = 0.22, weak effect size). Table 3 reveals no significant correlation between dental anxiety and toothbrush hardness preference. Medium hardness is most common; hardness is rare. Table 4 and Table 5 suggest no clear link between dental anxiety and flossing/mouthwash usage frequency. Notably, “never” and “once a day” were consistently favored for both practices.

### 3.2. Impact on Prophylaxis Appointments

In Table 6, most participants opt for annual calculus removal (N = 612), followed by twice a year (N = 300). Those who never get calculus removed have the highest mean DAS score (M = 10.85). As removal frequency rises, mean DAS scores generally decrease, notably from M = 10.85 (“never”) to M = 9.48 (“once a year”). Welch-Anova with Games-Howell post-hoc reveals a significant mean DAS difference between the “never” group and those with yearly removal with an effect size of eta^2^ = 0.019. Slight decreases in higher frequency removal were nonsignificant. Regarding “Professional Teeth Cleaning” appointments (Table 7), twice as many choose special appointments. The Welch test indicates highly significant DAS score differences between groups (M = 9.31 for “yes” and M = 10.19 for “no”), with a small effect (d = 0.22). Examining DAS and OHIP-G5 scores correlation, N = 1372 participants’ mean OHIP-G5 scores were calculated across DAS scores 4 to 20. Spearman’s rank correlation (*p* < 0.001) confirms a significant rise in OHIP-G5 scores with increasing DAS scores. A medium positive effect (Spearman’s rho = 0.335) implies higher dental anxiety corresponds to poorer oral health-related quality of life.

## 4. Discussion

Dental anxiety has far-reaching implications that extend beyond its impact on oral health. It influences various aspects of life, including career opportunities, social interactions, and self-esteem [19,34]. Moreover, it leads to prolonged and more costly dental treatments, placing a burden on the affected person, the dentists, and the healthcare system as a whole [35,36].

In this study, a large sample from a German adult population was analyzed, and anxiety levels were measured using the Dental Anxiety Scale (DAS). The average DAS score in this study was found to be M = 9.44, SD = 3.75, which aligns with similar findings from other studies conducted nationally and internationally, including Germany M = 9.70, SD = 3.40, Switzerland M = 10.4, SD = 3.89, and Brazil M = 9.34, SD = 3.50 [37,38,39].

Among the participants, those who reported not brushing their teeth had significantly higher DAS scores M = 14.00, SD = 5.25 compared to the other groups. However, the decrease in the DAS average with more frequent tooth brushing was not found to be statistically significant. This observation aligns with existing literature, which indicates that individuals with severe dental treatment anxiety often practice poor oral hygiene and have a heightened vulnerability to oral diseases [23,40]. The choice of toothbrush bristle hardness does not appear to be affected by dental anxiety. Despite the anticipation that anxious individuals might prefer a soft toothbrush for gentler cleaning, the highest average DAS score was associated with medium bristle hardness. Interestingly, while the use of electric toothbrushes has been gaining popularity, with around 40% of the population adopting them [15], this study found that 45.88% of participants reported using electric toothbrushes. Notably, users of electric toothbrushes tended to exhibit lower levels of anxiety compared to those who opted for manual toothbrushes. This discrepancy might be linked to the fact that individuals with dental treatment anxiety often avoid stimuli associated with dental procedures, including certain sounds, smells, and actions [41,42]. It is plausible that an electric toothbrush, due to its appearance, handling, and vibrations, may be perceived as a stronger negative stimulus and is therefore consequently rejected by anxious people. We also explored a possible correlation between dental anxiety and the frequency of dental floss and mouthwash usage. It was anticipated that individuals with dental fear would exhibit similar patterns in the use of additional oral care aids as they did in brushing frequency. Surprisingly, the data did not indicate a significant difference in dental anxiety among groups with different frequencies of dental floss or mouthwash usage. Purely in terms of numbers, most individuals used both either daily or not at all. It would have been also plausible that anxious patients would use mouthwash more regularly to mask bad breath, which is directly associated with poor oral health [43]. However, a trend can be observed in relation to dental anxiety. Flossing, due to its time-consuming nature and requirement for attention, can be seen as a stronger trigger for dental anxiety. Based on this assumption and the avoidance behavior exhibited by anxious individuals, the highest DAS score was found among those who reject flossing altogether. Conversely, using mouthwash is a quick and simple task that effectively masks bad breath. This could explain why the highest DAS score for mouthwash usage was observed among those who use it on a daily basis. The study also explored preventive practices commonly carried out in dental practices. We hypothesized that individuals with dental treatment anxiety would undergo dental calculus removal less frequently and decline professional teeth cleaning indicating an avoidance behavior. The majority of participants underwent dental calculus removal once a year, aligning with recommended annual check-ups. In Germany, dental calculus removal during these check-ups is covered by insurance, removing financial barriers. Participants who reported never undergoing dental calculus removal had the highest DAS scores, with an average of M = 10.85, SD = 4.52. In contrast, those who had it removed once or twice a year had DAS scores closer to the study’s average M = 9.48/9.31, SD = 3.89/3.50, and the lowest scores were observed in the group that underwent dental calculus removal more than twice a year M = 8.51, SD = 3.35. Thus, the group that never received dental calculus removal differed significantly from the other groups. Similarly, individuals declining professional teeth cleaning showed a statistically significant DAS score difference compared to those scheduling appointments. Those who had professional teeth cleaning had a score close to the study’s average M = 9.31, SD = 3.70, while the rejection group scored higher at M = 10.19, SD = 4.05. Once again, these findings affirm with the reports, that anxious patients tend to avoid appointments, seeking treatment mainly in case of pain [44]. However, these appointments, particularly those involving minimal invasive procedures, are crucial for the timely identification of oral hygiene deficiencies and targeted education for improvement. These appointments can help detect or even prevent potential issues in advance [45]. 

In addition to the Dental Anxiety Scale (DAS), the study also assessed the participants’ oral health-related quality of life (OHIP-G5). The mean OHIP-G5 score was M = 3.11, SD = 3.59, which is comparable to findings from other studies [46,47,48]. There was a highly statistically significant correlation between DAS scores and OHIP-G5 scores, indicating that higher dental anxiety was associated with poorer perceived oral-related quality of life. Participants with a DAS score of 11 or higher rated their oral health-related quality of life lower than the average. This threshold marked a significant deterioration in oral health-related quality of life. The effect size of eta^2^ = 0.149 for this correlation can be classified as strong. Daily at-home oral hygiene practices are one of the most effective means of maintaining oral health [49].

In conclusion, dental treatment anxiety remains a significant issue that negatively affects various dental preventive measures. In accordance with our hypotheses, it could be shown that dental anxiety is linked to lower frequencies of brushing, calculus removal and professional teeth cleaning. Furthermore, people tend to refrain from using an electric toothbrush and perceive their oral-health-related quality of life as decreasing with increasing dental fear. However, additional oral hygiene measures, such as the use of dental floss and mouthwash as well as the type of toothbrush, appear unaffected by dental anxiety and our hypotheses had to be dismissed. Avoidance behaviors stemming from dental anxiety not only lead to inadequate oral hygiene practices but also result in missed opportunities for preventive care, further exacerbating dental problems. Although dental clinics would be ideal environments for increasing awareness, anxious individuals tend to avoid such locations. Therefore, it is crucial to introduce targeted prevention programs starting from early childhood in order to alleviate fears and potentially prevent the development of dental anxiety.

It is essential to acknowledge the study’s limitations. In a cross-sectional design, causality cannot be determined. The analyzed data were self-reported by the patients and not objectively determined, thus the results cannot be generalized. The participants’ responses in anonymous surveys may still be influenced by societal pressure to provide socially desirable answers. Further limitations are that demographics and their influence were not addressed in this study. Also, the fact that just one basic anxiety scale was used which is not suitable for further distinguishing dental fear. For future research, an even higher sample size, categorized age groups, gender, marital status and education differences, and additional anxiety scales like the modified DAS or Dental Fear Survey should be considered to expand the current findings. 

## Figures and Tables

**Table 1:** Means of anxiety levels from different tooth brushing behavior.

What Is the Frequency of Your Daily Tooth Brushing?	N	DAS ScoreM (SD)	Anova
never	6	14.00 (5.25)	F(3) = 4.60, *p* = **0.003**, Eta^2^ = 0.08
once	229	9.97 (4.05)	
twice	1325	9.38 (3.73)	
three times or more	157	9.28 (3.72)	

**Table 2:** Means of DAS score of electric toothbrush users.

Do You Use an Electric Toothbrush?	N	DAS ScoreM (SD)	*t*-Test
yes	228	7.78 (2.97)	t(495) = 2.42, *p* = **0.016**, d = 0.22
no	269	8.48 (3.39)	

**Table 3:** Different anxiety levels compared to the hardness of the used toothbrush.

What Is the Level of Hardness of Your Toothbrush?	N	DAS ScoreM (SD)	Anova
soft	33	8.55 (3.37)	F(2) = 0.92, *p* = 0.401, Eta^2^ = 0.010
medium	139	9.24 (3.83)	
hard	13	8.08 (3.48)	

**Table 4:** Frequency of flossing compared to anxiety level means.

How Often Do You Floss Your Teeth?	N	DAS ScoreM (SD)	Anova
never	170	8.52 (3.27)	F(3) = 2.20, *p* = 0.088, Eta^2^ = 0.014
once a week	57	7.40 (2.70)	
once a day	207	7.95 (3.07)	
twice a day or more	51	8.31 (3.43)	

**Table 5:** Frequency of mouthwash usage compared to anxiety level means.

How Often Do You Use Mouthwash?	N	DAS ScoreM (SD)	Anova
never	224	8.00 (3.25)	F(3) = 1.76, *p* = 0.154, Eta^2^ = 0.011
once a week	49	7.51 (2.81)	
once a day	153	8.58 (3.06)	
twice a day or more	71	8.25 (3.64)	

**Table 6:** Frequency of calculus removal compared to DAS means.

How Often Do You Get Calculus Removed?	N	DAS ScoreM (SD)	Welch-Anova
never	152	10.85 (4.52)	F(3145.82) = 5.76, *p* < **0.001**, Eta^2^ = 0.019
once a year	612	9.48 (3.89)	
twice a year	300	9.31 (3.50)	
three times a year or more	35	8.51 (3.35)	

**Table 7:** Comparison of anxiety level and utilizing professional teeth cleaning appointments.

Do You Make Appointments Forprofessional Teeth Cleaning?	N	DAS ScoreM (SD)	Welch-Test
yes	893	9.31 (3.70)	t(699.40) = 3.681, *p* < **0.001**, d = 0.22
no	396	10.19 (4.05)	

## Data Availability

The data from this study were part of the dissertation paper from C.H.W. Data can be seen in Table 1, Table 2, Table 3, Table 4, Table 5, Table 6 and Table 7.
